# Retraction: Atrial septal defect repair; our early and mid-phase results

**Published:** 2014

This retracts the article “Atrial septal defect repair; our early and mid-phase results “ in Vol.30 No.2 from page 322-325, which was published in final edited form because of ethical misconduct as one of the authors copied some portion from an article published in Turkish language which was detected later. The authors have been notified and agree with the retraction. Retracted on April 15, 2014.

Ozcan S, Yener AU, Ozkan MTA. Atrial septal defect repair; our early and mid-phase results. Pak J Med Sci. 2014;30(2):322–325.

doi: http://dx.doi.org/10.12669/pjms.302.4759

